# Dose error reduction software in medication safety risk management – optimising the smart infusion pump dosing limits in neonatal intensive care unit prior to implementation

**DOI:** 10.1186/s12887-022-03183-8

**Published:** 2022-03-08

**Authors:** Sini Kuitunen, Krista Kärkkäinen, Carita Linden-Lahti, Lotta Schepel, Anna-Riia Holmström

**Affiliations:** 1 HUS Pharmacy and HUS Children and Adolescents, Helsinki University Hospital, Helsinki, Finland; 2 Division of Pharmacology and Pharmacotherapy, Faculty of Pharmacy, University of Helsinki, Helsinki, Finland; 3 HUS Pharmacy, Helsinki University Hospital, Helsinki, Finland; 4 HUS Joint Resources and HUS Pharmacy, Helsinki University Hospital, Helsinki, Finland

**Keywords:** Dose error reduction software, Error reporting, High-alert medication, Medication error, Medication safety, Neonatal intensive care unit, Proactive risk management, Smart infusion pump

## Abstract

**Background:**

Smart infusion pumps with dose error reduction software can be used to prevent harmful medication errors. The aim of this study was to develop a method for defining and assessing optimal dosing limits in a neonatal intensive care unit’s smart infusion pump drug library by using simulation-type test cases developed based on medication error reports.

**Methods:**

This mixed-methods study applied both qualitative and quantitative methods. First, wrong infusion rate-related medication errors reported in the neonatal intensive care unit during 2018–2019 were explored by quantitative descriptive analysis and qualitative content analysis to identify the error mechanisms. The researchers developed simulation-type test cases with potential errors, and a literature-based calculation formula was used to set upper soft limits to the drug library. The limits were evaluated by conducting programming of pumps without errors and with potential errors for two imaginary test patients (1 kg and 3.5 kg).

**Results:**

Of all medication errors reported in the neonatal intensive care unit, 3.5% (*n* = 21/601) involved an error or near-miss related to wrong infusion rate. Based on the identified error mechanisms, 2-, 5-, and 10-fold infusion rates, as well as mix-ups between infusion rates of different drugs, were established as test cases. When conducting the pump programming for the test cases (*n* = 226), no alerts were triggered with infusion rates responding to the usual dosages (*n* = 32). 73% (*n* = 70/96) of the erroneous 2-, 5-, and 10-fold infusion rates caused an alert. Mix-ups between infusion rates triggered an alert only in 24% (*n* = 24/98) of the test cases.

**Conclusions:**

Simulation-type test cases can be applied to assess the appropriateness of dosing limits within the neonatal intensive care unit’s drug library. In developing the test cases, combining hospital’s medication error data to other prospective data collection methods is recommended to gain a comprehensive understanding on mechanisms of wrong infusion rate errors. After drug library implementation, the alert log data and drug library compliance should be studied to verify suitability of dosing limits.

**Supplementary Information:**

The online version contains supplementary material available at 10.1186/s12887-022-03183-8.

## Background

Medication errors (MEs) are common in pediatric inpatient populations, and the risk of potential adverse drug events is significant in neonates, particularly in neonatal intensive care units (NICUs) [1–3]. Neonates are exposed to a higher risk of harm from MEs because of their rapidly changing body size and physical development, challenges to communicate with care providers, and more limited internal reserves to compensate for errors [4–6]. Also, the medication-use process in NICU is particularly complex because of the wide use of intravenous (IV) administration routes, weight-based small dosages, multiple calculations and dilutions, common off-label use, and the use of unlicensed drugs [2, 7–9]. MEs resulting in 10-fold, 100-fold, and even 1000-fold overdoses have been reported within the NICU settings, while such large deviations from the intended dose are less common in adult populations [10–14]. Moreover, many intravenously administered high-alert medications, such as opioids, insulin, vasoactive drugs, and parenteral nutrition, are used in NICU settings [2, 3, 7, 15]. As high-alert drugs bear a heightened risk for harm when used in error, proactive risk management strategies should be used to optimise these medication-use processes of neonatal patients [7, 15, 16].

The manual adjustment of infusion rates for each patient has been identified as an especially high-risk task of the IV medication-use process [7, 11, 17]. Smart infusion pumps with dose error reduction software (DERS) and associated drug libraries are designed to provide users with decision support in order to identify programming errors before starting the infusion [18]. Drug specific dosing limits can be placed to prevent both overdosing (upper limits) and underdosing (lower limits). While soft limits are intended to advise the user of potential errors and can be overridden, hard limits force functions to ensure that the facility-established medication-specific parameters are not exceeded. In the literature, unnecessary alerts resulting from poorly chosen dosing limits have been reported to contribute to alert fatigue among healthcare professionals using smart infusion pumps [18–25]. As a result, new medication safety risks arise from insufficient compliance in drug library use, and high override rates of soft limits have been identified. Other barriers to optimize the use of smart infusion pumps include limitations in pump capabilities, availability of pumps, programming workflow, associated risks with secondary infusions, pump data analysis, and deficiencies related to drug library use and updates (e.g., omitting certain drugs or IV fluids) [18, 26, 27].

To maximise the benefits of smart infusion pumps as a systemic defence and risk-reduction strategy, the dosing limits should be carefully optimised for each drug, patient group and care area before implementation [16, 18, 19, 21, 22, 28–31]. However, the scientific evidence about suitable methods for optimising the dosing limits in any patient care setting prior to their implementation in drug libraries is currently limited. The studies related to smart infusion systems mainly focus on the assessment of drug library compliance among caregivers, rate of triggered alerts, and soft limit override rates by using retrospective data collected from smart pump records [19, 22]. Also, studies exploring smart pumps in NICU [21] or wider hospital settings treating neonates [23–25, 32, 33] have focused on describing the building of drug libraries in a general level, and retrospectively evaluating drug library compliance or triggered alerts. The principles for setting dosing limits prior to implementation have not been described in detail. Overall, the evidence related to implementing smart infusion systems in the NICU settings is limited [21]. Therefore, the aim of this study was to develop a method for defining and assessing the optimal dosing limits in a NICU drug library. First, the utility of reported MEs in developing test patient cases to simulate potential programming errors was explored. Second, the alerts caused by the test cases were investigated to conclude the optimal dosing limits of the drug libraries.

## Methods

### Study design

**Fig. 1:**
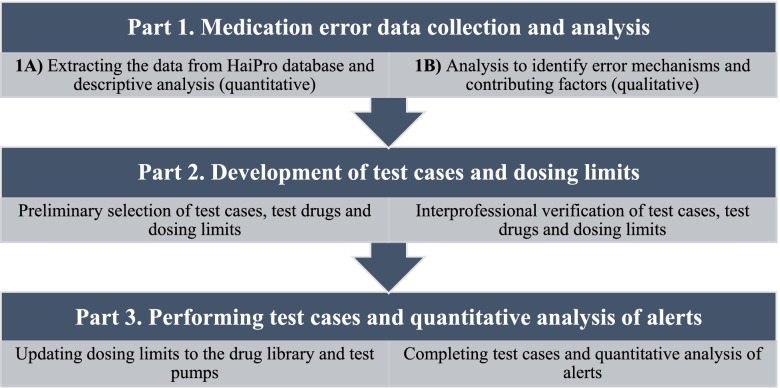
Flowchart of the study

This study was a mixed-methods investigation employing both quantitative and qualitative research methods [34]. The study was divided into three parts (Fig. 1), starting with quantitative and qualitative analysis of register-based NICU ME reports related to wrong infusion rate (Part 1) [16, 35, 36]. In the second part, the results of the ME analysis were utilised to create simulation-type test cases with potential errors, and dosing limits were set to a test sample of selected high alert medications in the NICU drug library [29, 37]. Finally, the test cases helped evaluate the appropriateness of the dosing limits quantitatively, including both right programming and potential errors. The study was based on the systems approach to risk management theoretical framework [16, 31].

### Study setting

The study took place in the NICU in Helsinki University Hospital (HUS), Finland, in 2020. The NICU has 29 registered beds and round-the-clock preparedness to receive and treat premature and full-term neonates in need of intensive care. Because most medications are used off-label, the approved use of every drug is described in the internal NICU medication guidelines. Perfusor Space (B. Braun Melsungen AG) syringe infusion pumps are used to administer all IV infusions in the unit. Before this study, the first version of the NICU drug library, including therapy groups to help drug selection, generic names, and standard concentrations, was customised with B. Braun Space OnlineSuite software (AP 2.1.2) and implemented in November 2019. The customisation was performed by a pediatric clinical pharmacist (SK) as a collaborative effort with a neonatologist, neonatal nurse practitioners, and a medication safety officer (CL-L, LS). This study was a part of the commissioning and programming of the drug library within B. Braun Space system in HUS NICU.

### ME data collection and analysis (part 1)

In the first part of the study, the NICU ME reports related to wrong infusion rates were explored to identify possible mechanisms behind these errors (Fig. 1) [16]. The data was extracted from HaiPro, a voluntary and anonymous electronic reporting system for patient- and medication-safety incidents largely used in Finland [38, 39]. In HUS, it was introduced in 2007 and extended to all departments in 2011. All hospital staff members can submit the reports. The reports comprise both structured- and open-narrative information on errors, which responsible persons (usually a senior doctor and an assistant head nurse) trained for the task then code in the units according to a certain structured classification system. MEs and near-misses reported in the NICU during 2018–2019 were extracted from the HaiPro database (LS). The reports related to wrong infusion rates were manually searched from the data by two researchers (KK, SK) independently. Only incidents identified by both researchers were included in the final research data. Disagreements on inclusion or exclusion of the error cases were resolved through discussion and consensus with a third researcher (A-RH).

The included ME reports were analysed both quantitatively and qualitatively (Fig. 1, Part 1). Quantitative descriptive analyses reporting frequencies and percentages were performed to the structured data (Fig. 1, Part 1A). The data comprised the medication involved in the error, event nature (e.g., ME or a near miss), event type (e.g., prescribing error, administration error), the consequences to the patient, consequences to the unit and the risk classification. In HaiPro system, the risk classification of ME reports is determined on a scale of I to V (I = insignificant risk, II = low risk, III = moderate risk, IV = significant risk, and V = serious risk). The risk classification is based on the combination of: 1) consequences of the injury to the patient (I = very minor, II = minor, III = moderate, IV = significant, V = severe) and 2) likelihood of error recurrence (I = rare, II = unlikely, III = possible, IV = probable, V = almost certain). Risk classification is used for identifying events posing a high risk to medication safety for further analysis in the healthcare organization using HaiPro. The researchers reviewed the original classification of the quantitative data (KK, SK) and corrected, if necessary. In addition, the Institute for Safe Medication Practices (ISMP) high-alert medications [15] involved in the ME reports were identified. The ISMP’s acute care list was chosen because it is widely used internationally and has also been applied in NICU settings [7].

The abductive qualitative content analysis was conducted to the open narrative data of ME reports to identify and categorise more specific error types, error mechanisms, and contributing factors (Fig. 1, Part 1B; Supplementary file 1) [16, 35, 36, 40]. Two researchers (SK, KK) analysed the narratives from systems approach to gather a more comprehensive understanding of the predefined issues (more specific error type, error mechanism, and contributing factors) associated with NICU MEs related to wrong infusion rates (Supplementary file 1). These predefined issues were used as main categories of data in the analysis. The findings were coded, and more specific sub-categories were generated based on the data. The size of the deviation from the intended dose was assessed when possible in the case of an overdose.

### Development of test cases and dosing limits (part 2)

In the second part of the study, test cases to optimise dosing limits were constructed by two researchers (KK, SK) based on the results of the ME analysis (Fig. 1, Part 2). The identified ME mechanisms applicable to continuous infusions were utilised to develop test cases. The drugs selected for the cases that occurred in the analysed ME reports were ISMP high alert drugs and were typically used in the NICU setting [15]. In addition, these drugs have been identified prone to pump-programming errors in other studies exploring smart infusion pumps in NICU and pediatric intensive care unit (PICU) settings [21, 23, 25]. Preliminary upper soft limits for each test drug were defined by multiplying the highest usual doses by 1.1, as this coefficient allows the prescriber to round doses [29]. Moreover, a 10% deviation of the reference dosage range has been identified as a dosing error threshold in PICU settings, as the evidence regarding NICUs remains limited [41].

In the HUS NICU, most continuous high-alert drug infusions are prescribed electronically in weight-based units (e.g., μg/kg/h), and the electronic health record (EHR) calculates the infusion rate (mL/h) by utilising the standard concentration (e.g., μg/mL) and patient’s weight (kg). However, there may be a need to exceed the usual maximum dose in exceptional cases in intensive care. Therefore, only overridable soft limits were decided to be used in the study. According to previous studies in PICU settings where patients range from neonates to adolescents, it is particularly important to set weight-based dosing limits [24]. The soft upper limits were placed in the drug library for each standard concentration in the same weight-based units as the drug is prescribed, and the patient’s weight is entered into the pump before programming the infusion rate (mL/h). The identified error mechanisms, test cases, imaginary patients, drugs and dosing limits were carefully reviewed and applied for the NICU’s clinical practice by the research group, neonatologist, and neonatal nurse practitioners before proceeding to part 3 of the study.

### Performing test cases and quantitative analysis of alerts (part 3)

In the last part of the study, the soft upper limits were loaded to the test pumps (Fig. 1, Part 3). Two researchers (SK, KK) individually programmed the pumps simultaneously to verify flawless programming (one repetition/test case/researcher), first with the usual doses of the test drugs to ensure that there are no alerts without an error. After that, the pumps were programmed according to the error-containing test cases when alerts were desirable (one repetition/test case/researcher). Since the objective was to demonstrate whether there was an alert or not associated with each test case, there was not seen a need for larger number of repetitions. The resulting alerts were documented and analysed by descriptive statistics (frequencies, percentages) to determine the appropriateness of the soft upper limits.

Study approval was obtained from the Helsinki University Hospital Joint Authority Administration. A separate ethics committee approval was not sought as the study did not contain any patient information or real patients.

## Results

### Quantitative analysis of ME reports (part 1A)

Altogether, 601 ME reports were submitted in HUS NICU during 2018–2019. Of all NICU ME reports, 3.5% (*n* = 21/601) involved an error or near-miss related to the wrong infusion rate. Characteristics of these ME reports are described in Table 1. Over half of the ME reports (*n* = 13/21) involved ISMP high-alert medications (*n* = 15), comprising fentanyl (*n* = 3), norepinephrine (*n* = 3), insulin (*n* = 3), parenteral nutrition (*n* = 2), heparin (*n* = 2), milrinone (*n* = 1), and dopamine (*n* = 1).

**Table 1:** Characteristics of wrong infusion rate related medication errors (ME) (*n* = 21)

Characteristic	Class	***n*** (%)
Error nature	Medication error	20 (95%)
	Near-miss	1 (5%)
Error type	Administration error	16 (76%)
	Prescribing error	4 (19%)
	Documenting error	1 (5%)
Harm to patient	Moderate harm	2 (10%)
	Minor harm	14 (66%)
	No harm	3 (14%)
	Not reported	2 (10%)
Harm to the unit	Additional work or minor procedures	20 (95%)^a^
	Additional costs	2 (10%)^a^
Risk classification^b^	Moderate risk (III)	15 (71%)
	Minor risk (II)	6 (29%)

Only classes occurring in the Neonatal intensive care unit’s ME reports are presented

^a^ One ME report was classified into two different classes/categories

^b^ Risk classification is determined in the organisation’s incident reporting system (HaiPro) on a scale of I to V according to the severity of the injury and the likelihood of error recurrence

### Qualitative analysis on ME reports (part 1B)

**Fig. 2:**
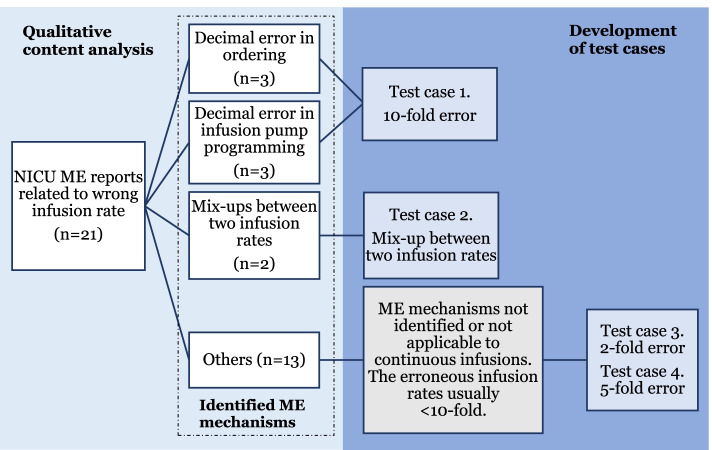
Development of medication error (ME) containing test cases (1-4) for test cases simulating infusion pump programming errors. The cases were invented based on ME mechanisms identified in a neonatal intensive care unit (NICU) ME reports (*n* = 21)

An error mechanism was identified in more than half of the cases (*n* = 11/21) (Fig. 2). These mechanisms were categorised based on data in six classes, which were a decimal error in ordering (*n* = 3), a decimal error in infusion pump programming (*n* = 3), mix-ups between two infusion rates (*n* = 2), a mix-up between dose (mg) and infusion rate (mL/h) of an intermittent infusion (*n* = 1), pausing the wrong infusion (*n* = 1) and a communication error related to dose change (*n* = 1). In the remaining ME reports (*n* = 10), the open narrative did not contain a sufficient case description, which is why the error mechanism could not be identified. In most cases (*n* = 15/21), the MEs led to an overdose, of which the largest deviation from the intended dose was 12-fold. Of the identified decimal errors (*n* = 6), 5 led to 10-fold infusion rate (e.g., norepinephrine infusion prescribed 0.03 mL/h, but the pump programmed 0.3 mL/h) and one led to 0.1-fold infusion rate.

One or more contributing factors were identified in the qualitative analysis in almost all ME report narratives (*n* = 19/21), and they were classified into seven categories based on the data. The contributing factors were failures to double-check the infusion rate (*n* = 9), heavy workload (*n* = 8), communication problems (*n* = 4), interrupted drug administration (*n* = 4), the limited number of nurses authorised to administer IV drugs (*n* = 3), night shift (*n* = 3), and missing systemic defences related to ordering stage (e.g., order verification or dose-range checking in the clinical decision support system) (*n* = 3).

### Development of test cases and dosing limits (part 2)

The test cases developed based on the error mechanisms identified in Part 1B are presented in Fig. 2. Most of the identified error mechanisms (*n* = 8/11) were applicable to the test cases on continuous infusions, resulting in test cases 1 (10-fold error) and 2 (mix-up between two infusion rates) (Fig. 2). As some of the error mechanisms (*n* = 3) did not apply to continuous infusions, and in some ME reports the error mechanism could not be identified (*n* = 10), the test cases 3 (2-fold error) and 4 (5-fold error) were developed based on the ME reports simulating smaller deviations than 10-fold errors from the intended doses. The selected test sample of high alert medications and their standard concentrations, usual dosages and drug library upper soft limits are presented in Table 2. Because of the wide size variation between NICU patients, the test cases were decided to be performed with two different sized imaginary test patients (1 kg and 3.5 kg).

**Table 2:** Test drugs, usual dosages and drug library soft upper limits used in the study

Drug	Standard concentration	Usual dosage	Soft upper limit
Fentanyl	5 μg/ml 10 μg/ml	0.5–1 μg/kg/h < 37 GA 0.5–2 μg/kg/h ≥ 37 GA	2.2 μg/kg/h
Norepinephrine	40 μg/ml	0.1–0.2–0.4 (≥ 0.5)^a^ μg/kg/min	0.55 μg/kg/min
Dopamine	1 mg/ml 2 mg/ml	2–5–10 (− 15)^a^ μg/kg/min	16.5 μg/kg/min
Heparin flush	0.6 IU/ml	0.36–0.6 IU/h	0.66 IU/h

*GA* gestational age

^a^The rarely used highest possible usual doses of norepinephrine and dopamine, which directed the establishing of dosing limits, but were not used in test cases and are presented in parentheses

### Performing test cases and quantitative analysis of alerts (part 3)

The results of the test cases (*n* = 226) are presented in Tables 3 and 4. Each test case was performed by two authors (SK, KK) independently without any observed errors in the programming of test cases. As expected, there were no alerts in test cases simulating usual dosages (*n* = 32) (Table 3). The soft upper limits caused an alert in 73% (*n* = 70/96) of test cases containing 2-fold, 5-fold and 10-fold errors. The 10-fold errors caused an alert in all test cases (*n* = 32). In the case of 2-fold and 5-fold errors, some of the lowest usual dosages did not cause an alert, as they were smaller than the maximum dosages. In the case of heparin flush having a weight-independent fixed-dose, all error scenarios produced an alert.

**Table 3:** T﻿he results of test cases simulating usual doses (*n* = 32) and test cases including erroneous 10-fold, 5-fold and 2-fold infusion rates (*n* = 96)

	Patient 1.0 kg			Patient 3.5 kg		
**Fentanyl 5 μg/ml**	**0.5** **μg/kg/h**	**1 μg/kg/h**	**2 μg/kg/h**	**0.5 μg/kg/h**	**1 μg/kg/h**	**2 μg/kg/h**
**Soft upper limit (mL/h)**	**0.44**	**0.44**	**0.44**	**1.54**	**1.54**	**1.54**
Usual rate (mL/h)	0.1	0.2	0.4	0.35	0.7	1.4
10-fold rate (mL/h)	1.0^a^	2.0^a^	4.0^a^	3.5^a^	7.0^a^	14.0^a^
5-fold rate (mL/h)	0.5^a^	1.0^a^	2.0^a^	1.75^a^	3.5^a^	7.0^a^
2-fold rate (mL/h)	0.2	0.4	0.8^a^	0.7	1.4	2.8^a^
**Fentanyl 10 μg/ml**	**0.5 μg/kg/h**	**1** **μg/kg/h**	**2** **μg/kg/h**	**0.5 μg/kg/h**	**1 μg/kg/h**	**2 μg/kg/h**
**Soft upper limit (mL/h)**	**0.22**	**0.22**	**0.22**	**0.77**	**0.77**	**0.77**
Usual rate (mL/h)	0.05	0.1	0.2	0.18	0.35	0.7
10-fold rate (mL/h)	0.5^a^	1.0^a^	2.0^a^	1.8^a^	3.5^a^	7.0^a^
5-fold rate (mL/h)	0.25^a^	0.5^a^	1.0^a^	0.9^a^	1.75^a^	3.5^a^
2-fold rate (mL/h)	0.1	0.2	0.4^a^	0.36	0.7	1.4^a^
**Dopamine 1 mg/ml**	**2 μg/kg/min**	**5** **μg/kg/min**	**10 μg/kg/min**	**2** μg**/kg/min**	**5 μg/kg/min**	**10 μg/kg/min**
**Upper soft limit (mL/h)**	**0.99**	**0.99**	**0.99**	**3.46**	**3.46**	**3.46**
Usual rate (mL/h)	0.12	0.3	0.6	0.42	1.05	2.1
10-fold rate (mL/h)	1.2^a^	3.0^a^	6.0^a^	4.2^a^	10.5^a^	21.0^a^
5-fold rate (mL/h)	0.6	1.5^a^	3.0^a^	2.1	5.25^a^	10.5^a^
2-fold rate (mL/h)	0.24	0.6	1.2^a^	0.84	2.1	4.2^a^
**Dopamine 2 mg/ml**	**2 μg/kg/min**	**5 μg/kg/min**	**10 μg/kg/min**	**2 μg/kg/min**	**5 μg/kg/min**	**10 μg/kg/min**
**Upper soft limit (mL/h)**	**0.49**	**0.49**	**0.49**	**1.73**	**1.73**	**1.73**
Usual rate (mL/h)	0.06	0.15	0.3	0.21	0.53	1.05
10-fold rate (mL/h)	0.6^a^	1.5^a^	3.0^a^	2.1^a^	5.3^a^	10.5^a^
5-fold rate (mL/h)	0.3	0.75^a^	1.5^a^	1.05	2.65^a^	5.25^a^
2-fold rate (mL/h)	0.12	0.3	0.6^a^	0.42	1.06	2.1^a^
**Norepinephrine 40 μg/ml**	**0.1 μg/kg/min**	**0.2 μg/kg/min**	**0.4 μg/kg/min**	**0.1 μg/kg/min**	**0.2 μg/kg/min**	**0.4 μg/kg/min**
**Upper soft limit (mL/h)**	**0.82**	**0.82**	**0.82**	**2.89**	**2.89**	**2.89**
Usual rate (mL/h)	0.15	0.3	0.6	0.53	1.05	2,1
10-fold rate (mL/h)	1.5^a^	3.,0^a^	6.0^a^	5.3^a^	10.5^a^	21.0^a^
5-fold rate (mL/h)	0.75	1.5^a^	3.0^a^	2.65	5.25^a^	10.5^a^
2-fold rate (mL/h)	0.3	0.6	1.2^a^	1.06	2.1	4.2^a^
**Heparin flush 0.6 IU/ml**	**0.36 IU/h**	**0.6 IU/h**				
**Upper soft limit (mL/h)**	**1.11**	**1.11**				
Usual rate (mL/h)	0.6	1.0				
10-fold rate (mL/h)	6.0^a^	10.0^a^				
5-fold rate (mL/h)	3.0^a^	5.0^a^				
2-fold rate (mL/h)	1.2^a^	2.0^a^				

The pumps were programmed with the rate corresponding to the usual dose (*n* = 32) and erroneously with 10-fold, 5-fold and 2-fold infusion rates (*n* = 96). (^a^) identifies the test cases where the soft upper limit triggered an alert (*n* = 70/96, 73%)

**Table 4:** The results of mix-up test-cases when programming the pumps with another drug’s infusion rate (*n* = 98)

	Patient 1.0 kg			Patient 3.5 kg		
**Fentanyl 5 μg/ml**	**Soft upper limit 0.44 mL/h**			**Soft upper limit 1.54 mL/h**		
Dopamine 1 mg/mL (mL/h)	0.12	0.3	0.6^a^	0.42	1.05	2.1^a^
Norepinephrine 40 μg/ml (mL/h)	0.15	0.3	0.6^a^	0.53	1.05	2.1^a^
Heparin flush 0.6 IU/ml^a^ (mL/h)	0.6^a^	1.0^a^	N/A	0.6	1.0	N/A
**Fentanyl 10 μg/ml**	**Soft upper limit 0.22 mL/h**			**Soft upper limit 0.77 mL/h**		
Dopamine 2 mg/mL (mL/h)	0.06	0.15	0.3^a^	0.21	0.53	1.05^a^
Norepinephrine 40 μg/ml (mL/h)	0.15	0.3^a^	0.6^a^	0.53	1.05^a^	2.1^a^
Heparin flush 0.6 IU/ml^a^ (mL/h)	0.6^a^	1.0^a^	N/A	0.6	1.0^a^	N/A
**Dopamine 1 mg/ml**	**Soft upper limit 0.99 mL/h**			**Soft upper limit 3.46 mL/h**		
Fentanyl 5 μg/ml (mL/h)	0.1	0.2	0.4	0.35	0.7	1.4
Norepinephrine 40 μg/ml (mL/h)	0.15	0.3	0.6	0.53	1.05	2.1
Heparin flush 0.6 IU/ml^a^ (mL/h)	0.6	1.0^a^	N/A	0.6	1.0	N/A
**Dopamine 2 mg/ml**	**Soft upper limit 0.49 mL/h**			**Soft upper limit 1.73 mL/h**		
Fentanyl 10 μg/ml (mL/h)	0.05	0.1	0.2	0.18	0.35	0.7
Norepinephrine 40 μg/ml (mL/h)	0.15	0.3	0.6^a^	0.53	1.05	2.1^a^
Heparin flush 0.6 IU/ml^a^ (mL/h)	0.6^a^	1.0^a^	N/A	0.6	1.0	N/A
**Norepinephrine 40 μg/ml**	**Soft upper limit 0.82 mL/h**			**Soft upper limit 2.89 mL/h**		
Fentanyl 5 μg/ml (mL/h)	0.1	0.2	0.4	0.35	0.7	1.4
Dopamine 1 mg/mL (mL/h)	0.12	0.3	0.6	0.42	1.05	2.1
Heparin flush 0.6 IU/ml (mL/h)	0.6	1.0^a^	N/A	0.6	1.0	N/A
**Heparin flush 0.6 IU/ml**	**Soft upper limit 1.1 mL/h**			**Soft upper limit 1.1 mL/h**		
Fentanyl 5 μg/ml (mL/h)	0.1	0.2	0.4	0.35	0.7	1.4^a^
Dopamine 1 mg/mL (mL/h)	0.12	0.3	0.6	0.42	1.05	2.1^a^
Norepinephrine 40 μg/ml (mL/h)	0.15	0.3	0.6	0.53	1.05	2.1^a^

Regardless of the patient’s weight, heparin flush is always used in one of the two optional infusion rates. Therefore, only two test results are reported to mix-ups with heparin flush (N/A indicates no test result reported in the column)

(^a^) identifies the test cases where the soft upper limit triggered an alert (*n* = 24/98, 24%)

The test case regarding the mix-ups between two infusion rates (Table 4) was simulated by programming the pump with all other test drugs’ rates (Table 3). The higher standard concentrations of fentanyl and dopamine were cross-programmed with each other, as they are often simultaneously used with fluid restricted patients. The mix-ups caused an alert in 24% (*n* = 24/98) of test cases when the erroneous infusion rate was higher than the usual maximum dose (Table 4). The remaining mix-ups did not cause an alert because the erroneous dose was lower than the usual maximum dose.

## Discussion

To the best of our knowledge, this is the first study aiming to optimise drug library dosing limits in smart infusion pumps prior to their implementation in a NICU environment. Our research was based on the systems approach to preventive medication safety risk management stating that risks should be identified and managed proactively before they reach the patient [16]. The findings of our study support the use of hospitals’ own ME reports and the existing literature to identify risks associated with wrong infusion rate and to optimise drug library dosing limits as systemic defences before their implementation. Based on the NICU ME reports, we developed test cases to assess the dosing limits in the NICU infusion pump drug library; the test cases may also apply to other pediatric populations. However, the reliability of test cases could be developed further by using prospective data collection methods, such as direct observation, focus groups and interviews with practitioners to gain even more comprehensive understanding on mechanisms of wrong infusion rate errors within human factors framework [16, 31, 42–44]. Our results indicate that the literature-based calculation formula developed to define the soft upper limits in pediatric intensive care settings [29] seems to be applicable in NICU settings.

Our results are promising from the perspective of the widely reported risk of alert fatigue associated with poorly defined soft limits [19, 22]. As expected, the usual dosages did not cause any alerts in this study, while 10-fold errors triggered an alert in all test cases. One of the key factors that made this result possible was the contribution of the neonatologist in a careful assessment of the usual maximum doses of test drugs in collaboration with the research group. Earlier studies have reported clustering of dose error reduction software (DERS) alerts around specific medications and patients (e.g., fentanyl, vasopressin, and insulin in palliative care, when sedatives and analgesics have been significantly escalated) [21]. Therefore, it would be useful to target similar testing activities to these particular drugs and patient groups as presented in this study.

Our analysis of the ME reports related to wrong infusion rate resulted in similar findings to earlier studies in NICU settings. Most MEs involved a high-alert medication and resulted in overdoses [1, 2, 7, 11, 12, 32]. MEs can be difficult to identify before reaching the patient because of varying treatment and patient related factors, such as small drug doses and wide size variations between different patients. However, in the NICU settings, the drug library hard limits as system-based barriers have prevented administration of doses even as high as 29-fold compared to the maximum dose [16, 21, 31]. Especially when high-alert medications are involved, MEs with this size of deviations from the intended dose expose vulnerable NICU patients to serious adverse drug events [2, 7, 10–13, 15, 21]. Following earlier studies, our analysis of contributing factors to wrong infusion rate errors also revealed that failures in the use of other systemic defences or not having them implemented could enable errors [17]. Consequently, a combination of different preventive error reduction strategies is needed in IV medication use process to mitigate the effects of e.g., environmental, operational and team-work related factors on human performance [16, 19, 22, 31, 45].

We demonstrated that errors involving doses lower than the usual maximum dose could not have been avoided by using DERS (e.g., the smallest usual doses and most test cases involving a mix-up between two infusion rates). However, a bi-directional smart infusion pump interoperable with the EHR would provide such a solution for even more comprehensive management of human factors contributing to pump-programming errors due to manual adjustment of infusion rate [16, 18, 28, 31, 46]. The system would enable auto-programming of infusion parameters (e.g., infusion rate) from the EHR system to the pump, which are then verified and followed by starting the infusion by a practitioner [18]. The pump also automatically sends infusion information (e.g., dose-rate, rate changes, and IV start and stop times) to the EHR system for practitioner confirmation to enable accurate recording of this information in the patient’s record. However, as with smart infusion pumps, the introduction of interoperability with EHR has been associated with challenges, such as inadequate and outdated drug libraries, pump or medications not mapped with the EHR system, and inconsistency in dosing units between the drug library, EHR and usual pump-programming practices [44].

Our results support the use of weight-based dosing limits in NICU drug libraries, which has been reported as one of the key elements of pediatric drug libraries [24, 29]. As a result, all the most crucial programming errors (e.g., 10-fold infusion rate) triggered an alert. The test cases related to heparin flush demonstrated that when the medication does not require weight-dependent dosing, the drug library dosing limits are much easier to set. However, it should be noted that when smart pumps are used without EHR interoperability, patient’s weight needs to be entered into the pump when programming the infusion. This represents an additional manual step with a chance for human error [16, 31].

There are some limitations to the study. First, we used self-reported ME data to create test cases simulating errors resulting in the wrong infusion rate. Self-reporting is associated with the risk of underreporting, and it is unlikely that all errors and near-misses were documented [47, 48]. The number of ME reports included in qualitative content analysis remained low, as we focused only on one part of the medication use process, and neonates are a limited patient group. However, our aim was to study the possible error mechanisms contributing to wrong infusion rates, specifically in NICU settings instead of error incidence. Therefore, the self-reported ME data was found useful for the purpose of this study. To improve the reliability, two researchers independently searched ME reports meeting the inclusion criteria and verified the findings of the qualitative content analysis, followed by a careful review of the error mechanisms and test cases by the research group, neonatologist, and neonatal nurse practitioners. Nonetheless, qualitative content analysis is a researcher’s subjective interpretation. Some ME reports described the incidents only briefly, so the researchers’ interpretations might not entirely correspond to the actual incidents [35]. In future studies, the test cases should be further developed by using data collected through prospective methods and other theoretical frameworks, such as focus groups and SEIPS (Systems Engineering Initiative for Patient Safety) [43, 49].

Second, we only used soft upper limits even though an effective DERS should include hard and soft upper and lower dosing limits [18, 19, 22]. Earlier studies have reported a high override rate of soft limits, and therefore, all alerts triggered in our study cannot be equated as averted errors in clinical situations. However, not all pump-programming errors cause significant patient harm, which was found out in our ME analysis and has also been observed elsewhere [25]. Moreover, the number of medications selected to perform the test cases was relatively small, and the selection of different test drugs might have resulted in different findings. When it comes to demonstrating mix-ups between two drug’s infusion rates, the future studies should include designs enabling a more comprehensive exploration of environmental and team-work related factors (e.g., a simulation study with full patient scenarios and multiple end-user participants) [16, 31, 45].

The current study represents a preliminary work aiming to define dosing limits before their implementation, but the true effectiveness of these limits can be reliably evaluated only after implementation. In future studies, the alert log data and drug library compliance should be studied after implementation of dosing limits to confirm whether the limits have a beneficial effect on drug library compliance and soft limit alert overrides [18, 19, 22]. Also, a simulation study involving patient scenarios, real care teams and simulated care environments would be beneficial to examine the optimal use of both hard and soft limits [37]. However, the present study provides NICU and possibly other settings with means for targeting optimal dosing limits, as improperly defined hard limits can prevent legitimate actions. In contrast, unsuitable soft limits can cause useless alerts [19, 22].

## Conclusion

Simulation-type test cases can be successfully applied to assess the appropriateness of dosing limits within the NICU drug library. In developing the test cases, combining hospital’s medication error data to other prospective data collection methods is recommended to gain a comprehensive understanding on mechanisms of wrong infusion rate errors within the human factors framework. However, when the lowest usual drug doses are used, a larger deviation from the intended infusion rate is required to generate an alert. Consequently, combining smart infusion pumps to other systemic defenses in the IV medication use process is required for a more comprehensive preventive risk management approach. In future studies employing a similar method for defining and testing the dosing limits, the alert log data and drug library compliance should be studied post implementation to verify the suitability of the dosing limits.

## Supplementary Information

**Additional file 1: Figure**. A more detailed description of the abductive content analysis in Part 1B of the study.

## Data Availability

The HaiPro medication error report data that support the findings of this study are available from Helsinki University Hospital (HUS), but restrictions apply to the availability of these data, which were used under license for the current study, and so are not publicly available. Data are, however, available from the authors upon reasonable request and with permission of HUS.

## Abbreviations

DERS: Dose error reduction software
EHR: Electronic health record
HUS: Helsinki University Hospital
ISMP: Institute for Safe Medication Practices
IV: Intravenous
ME: Medication error
NICU: Neonatal intensive care unit
