# Supplementary material for: Dose error reduction software in medication safety risk management – optimising the smart infusion pump dosing limits in neonatal intensive care unit prior to implementation
Source: BMC Pediatr. 2022 Mar 8;22:118. doi: 10.1186/s12887-022-03183-8 (PMC8902762; doi:10.1186/s12887-022-03183-8)
Supplement: Supplementary file 1 — Additional file 1: Figure. A more detailed description of the abductive content analysis in Part 1B of the study. [file 12887_2022_3183_MOESM1_ESM.docx]

**Supplementary file 1. A detailed description of the abductive qualitative content analysis in Part 1B of the study.**

**Figure**. An example of the abductive qualitative content analysis. ME=medication error, IV=intravenous, NICU=neonatal intensive care unit.

**
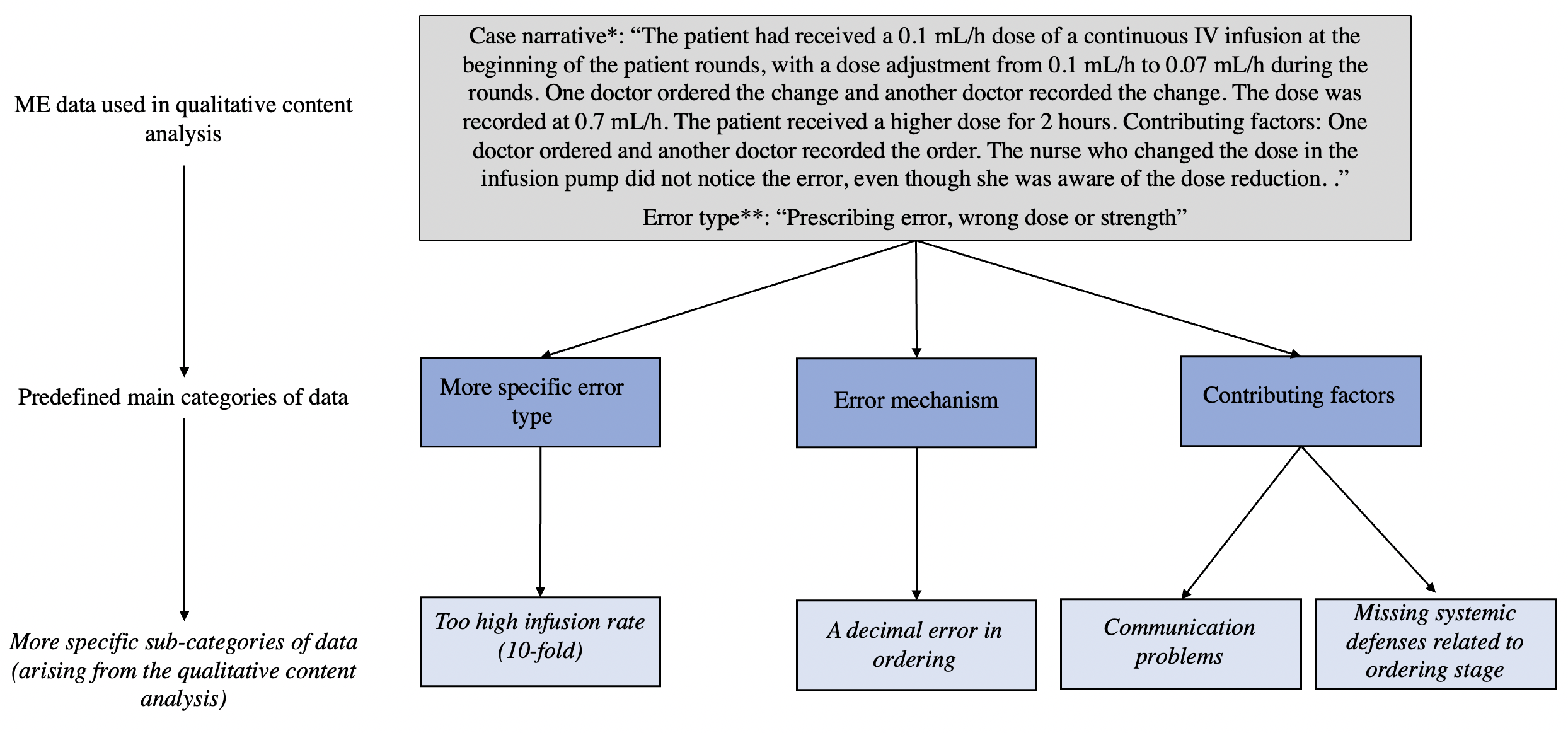
**

*The case narrative is presented in a shortened and anonymized format to ensure patient anonymity.
**Error type defined in the NICU by the persons responsible for handling the ME reports (usually a senior doctor and an assistant head nurse trained for the task according to structured HaiPro classification).

**Table**. Examples (n=5) of abductive qualitative content analysis of medication error (ME) reports (n=21). In addition to open case narratives, error type classified according to HaiPro classification system was used to support the analysis. In the first part of the analysis, the open case narratives of ME reports were searched to identify data related to predefined main categories (more specific error types, error mechanisms, and contributing factors). These findings are highlighted with yellow color in the shortened and anonymized case narratives. In the second part of the analysis, the findings were classified to more specific sub-categories arising from the data. The case narratives are presented in a shortened and anonymized format to ensure patient anonymity.

| **Open case narratives and classified HaiPro data used to support the abductive qualitative content analysis** | | **Predefined main categories of data** | | |
| --- | --- | --- | --- | --- |
| **Shortened and anonymized case narrative** | **Error type according to HaiPro classification** | **More specific error type** | **Error mechanism** | **Contributing factors** |
|  |  | ***More specific sub-categories of data (arising from the qualitative content analysis)*** | | |
| Continuous IV infusion started. Order 0.1 mL/h. The patient had received the infusion for 25 min, after we started wondering at the low infusion rate. Asked a doctor, there is an error in the order. Correct rate 1 mL/h. Contributing factors: Night shift. | Prescribing error: wrong dose or strength | *Too low infusion rate (0,1-fold)* | *A decimal error in ordering* | *Night shift* |
| The patient received a continuous IV infusion at 0.1 mL/h dose at the beginning of the patient rounds, with an adjustment from 0.1 mL/h to 0.07 mL/h during the rounds. One doctor ordered the change and another doctor recorded the change. The dose was recorded at 0.7 mL/h. The patient received a higher dose for 2 hours. Contributing factors: One doctor ordered and another doctor recorded the order. The nurse who changed the dose in the infusion pump did not notice the error, even though she was aware of the dose reduction. | Prescribing error: wrong dose or strength | *Too high infusion rate (10-fold)* | *A decimal error in ordering* | *Communication problems*  *Missing systemic defenses related to ordering stage* |
| A daily change of medication and fluid infusions. Patient with multiple drug infusions and an IV fluid to change. A continuous drug infusion prescribed at a rate of 0.03 mL/h, inadvertently set to go 0.3 mL/h. The nurse who started the infusion noticed the error herself (had not yet had time to double-check the fluids). Contributing factors: There are 2 children in the room. Lots of fluids / medications to change simultaneously, including arterial system change. | Administration error: wrong dose or strength | *Too high infusion rate (10-fold)* | *A decimal error in infusion pump programming* | *Failure to double-check the infusion rate*  *Heavy workload* |
| The patient had 1^st^ continuous IV infusion of 0.15 mL/h and 2^nd^ continuous IV infusion of 0.05 mL/h. When changing the IV infusions, the infusion rates were inadvertently reversed, i.e., 1^st^ IV infusion 0.05 mL/h and 2^nd^ 0.15mL/h. The patient received drug infusions at the wrong rate for two hours before the situation was noticed. Contributing factors: A busy day. The patient was about to have a sterile procedure and the infusions had to be changed before that. The infusion pump also “did a trick” and did not allow the desired rate of 0.05mL/h, by always increasing the rate to 0.1mL/h, so the pump had to be replaced. Infusions could not be double-checked right away, as the sterile procedure prolonged. The nurse was also responsible of another child in the room at the same time, which weakened concentration to this patient. | Administration error: wrong dose or strength | *Too high and too low infusion rate (3-fold)* | *A mix-up between two infusion rates* | *Failure to double-check the infusion rate*  *Heavy workload*  *Interrupted drug administration* |
| When starting an intermittent infusion, the pump was accidentally set at a rate of 20mL/h instead of the intended 4mL/h (patient's dose 20mg). This may have been because we were talking to the baby's parents at the same time and the focus on medication was not perfect. Because of this, the baby received the infusion in 40 minutes instead of 60 minutes. Contributing factors: Conversation with the parents at the same time possibly interfered with concentration to the task. | Administration error: wrong method of drug administration | *Too high infusion rate (5-fold)* | *Other (a mix-up between drug dose and infusion rate)* | *Interrupted drug administration* |
